# Beyond boundaries: extended temporal flexibility in synaptic tagging and capture

**DOI:** 10.1038/s42003-025-07998-w

**Published:** 2025-04-04

**Authors:** Yee Song Chong, Sheila Ruixia Ang, Sreedharan Sajikumar

**Affiliations:** 1 Department of Physiology, Yong Loo Lin School of Medicine, National University of Singapore, Singapore, Singapore; 2 Life Sciences Institute Neurobiology Programme, National University of Singapore, Singapore, Singapore; 3 Healthy Longevity Translational Research Programme, Yong Loo Lin School of Medicine, National University of Singapore, Singapore, Singapore

**Keywords:** Neuroscience, Learning and memory

## Abstract

Synaptic tagging and capture (STC) is a mechanism that enables the formation of associative synaptic plasticity by marking activated synapses with “tags” to capture plasticity-related products (PRPs) essential for plasticity stabilization. Experimental evidence using long-term potentiation (LTP), a widely studied cellular correlate of memory, shows that the duration of synaptic tags varies, lasting up to 90 minutes in ex vivo hippocampal slices but shorter in in vivo conditions, likely due to higher metabolic activity. In this study, we investigate the time window for tag-PRP interactions in STC using a strong-before-weak paradigm, where protein synthesis-dependent late-LTP precedes protein synthesis-independent early-LTP at various intervals. Surprisingly, successful STC is observed even with a 9-hour interval in the strong-before-weak paradigm, suggesting a broader temporal flexibility for tag-PRP interactions than previously understood. This unexpected finding offers alternative explanations for associative memory formation by cataloguing memory events, allowing weaker memories to be strengthened when preceded by stronger ones.

## Abstract (web summary)

Ex vivo synaptic tagging and capture allows tag–Plasticity-Related Protein (PRP) interactions to persist for over 9 hours—far longer than previously thought—offering new insights into associative memory formation and the temporal flexibility of memory processes.

## Introduction

New information is continuously received and processed in the brain every day, every moment. The brain has mechanisms to filter out irrelevant details and retain useful information, enhancing its efficiency. However, when seemingly irrelevant details are linked to memorable experiences or occur in temporal proximity to significant events, the brain may still reinforce these details, transforming them into long-lasting memories. This remarkable capacity of the brain to connect related memories is known as associativity. At the cellular level, associativity is also observed in long-term potentiation (LTP), a well-studied form of synaptic plasticity widely recognized as a cellular correlate of learning and memory^1^ (for a review, see Bin Ibrahim, Benoy, & Sajikumar, 2022)^2^.

The associative property of LTP is elegantly exemplified by the synaptic tagging and capture (STC) model^3,4^. According to the STC model, the maintenance of LTP involves distinct processes, including the setting of tags to capture plasticity-related products (PRPs) and the synthesis of PRPs, which can be initiated by various stimuli. Tag setting, or “tagging,” represents a temporary state of activated synapses that primes them to capture available PRPs. Newly synthesized PRPs are distributed throughout the neuron but are only utilized by tagged synapses. Frey and Morris^3^ demonstrated the STC model by showing that an LTP event, which does not independently trigger PRP synthesis, can nevertheless be sustained at a potentiated level if PRPs are available from another source. Further investigations into the STC model paired strong tetanization-induced late-LTP with a weak tetanization event that typically induces protein synthesis-independent early-LTP^5–23^. In this context, the early-LTP was transformed into long-lasting LTP, suggesting that tag setting and PRP synthesis are two independent processes. The timing of tag setting and PRP synthesis is essential for synaptic associativity. If PRPs are unavailable while tagging is active, the potentiation of LTP will eventually decay. This dependency was demonstrated in previous studies using a “weak-before-strong” STC paradigm, where different conditions exhibited varying tagging durations. In CA1 ex vivo hippocampal slices, tag setting persisted for up to 90 minutes but could be extended through metaplasticity^6,11^. By contrast, in vivo field recordings and behavioural tagging showed shorter durations for tag setting^24^, likely due to intact modulatory systems in the in vivo environment, which increase the metabolic rate. In contrast, little is known about how the duration of newly synthesized PRPs affects STC. It stands to reason that LTP associativity or memory formation should occur within a limited temporal window, maintaining the brain’s high energy efficiency.

In this study, we aimed to investigate the time window for tags-PRPs interaction in STC using ex vivo extracellular electrophysiological field recording technique. We used strong-before-weak and weak-before-strong STC paradigms with varied intervals. Surprisingly, we observed successful STC even when the STC interval was increased to 9 h in the strong-before-weak paradigm.

## Methods

### Animals and hippocampal slices preparation

A total of 83 transverse hippocampal slices from 55 male C57BL/6J mice at 5–7 weeks old were used in this study. Female mice were excluded to avoid possible hormonal alterations during the oestrous cycle that can affect synaptic plasticity measurements^25–27^. Animals were purchased from InVivos Pte Ltd (Singapore). The animals were housed under 12 h light/dark cycle with food and water provided *ad libitum*. All experimental procedures were approved by the Institutional Animal Care and Use Committee (IACUC) of the National University of Singapore. We have complied with all relevant ethical regulations for animal use.

Animals were anaesthetised using CO_2_ and then decapitated after cervical dislocation. The brain was quickly transferred into ice-cold (4 °C) artificial cerebrospinal fluid (aCSF) saturated with carbogen (95% O_2_ and 5% CO_2_). The composition of aCSF was as followed (in mM): 124.0 NaCl, 3.7 KCl, 1.0 MgSO_4_·7H_2_O, 2.5 CaCl_2_·2H_2_O, 1.2 KH_2_PO_4_, 24.6 NaHCO_3_, and 10.0 D-glucose. The hippocampi were isolated and sliced into 400 μm thick transverse slices using Stoelting tissue slicer. Then, the hippocampal slices were placed onto a nylon net in an interface chamber (Scientific System Design) perfused with carbogenated aCSF at a rate of approximately 1–2 ml/min at 32 °C and preincubated for at least 3 h to achieve metabolic stability^28,29^. In all our experiments, slices were incubated for at least 2.5 hours before electrode positioning for extracellular field potential recordings. After electrode placement, slices were left for approximately 30 minutes before baseline recordings commenced, resulting in a total incubation time of 3 hours.

### Electrophysiological field recording

**Fig. 1:**
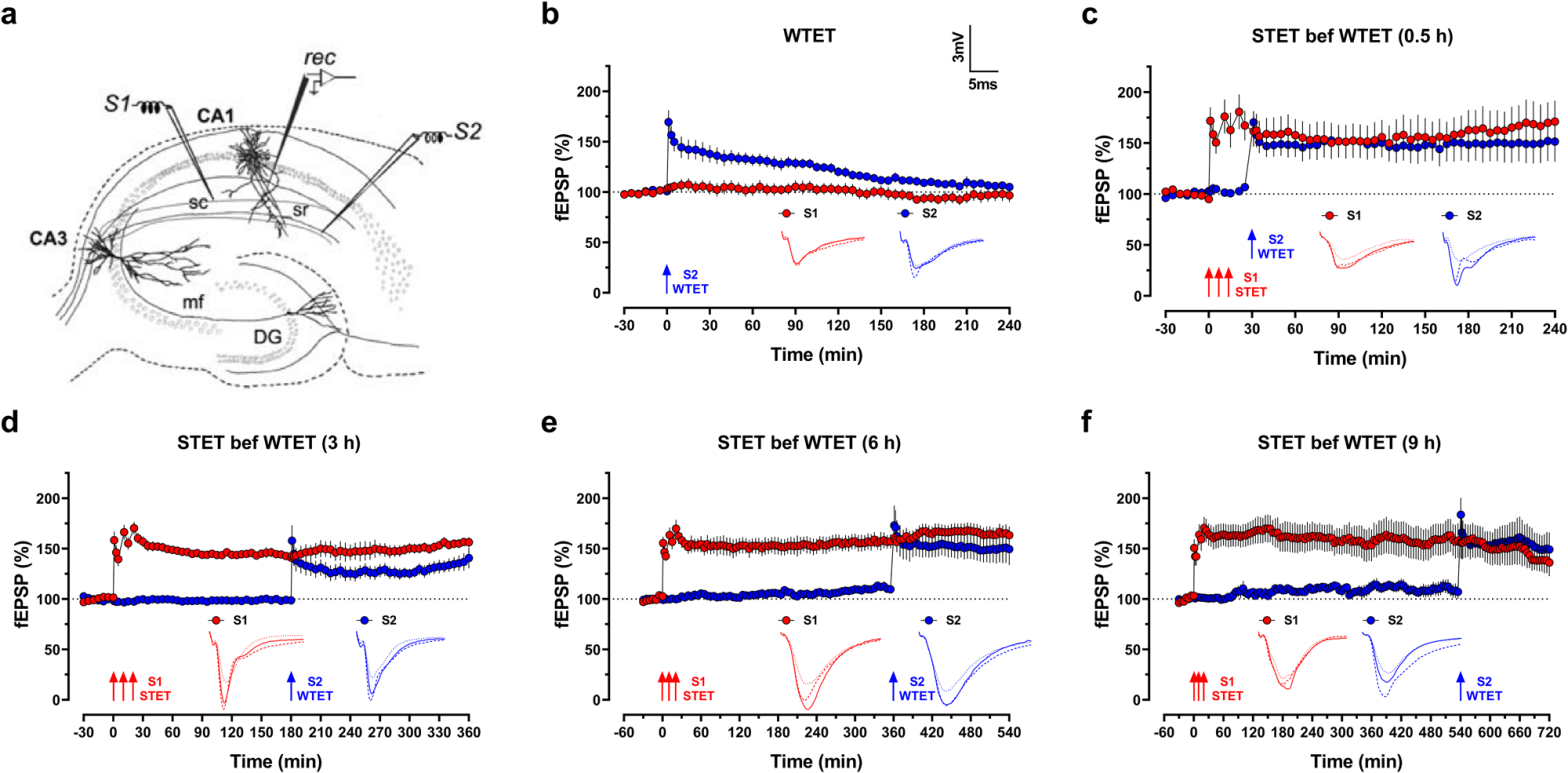
Extended associativity in strong-before-weak synaptic tagging and capture paradigm. **a** Schematic representation of a transverse hippocampal slice showing the location of electrodes in the CA1 region. Recording electrode (rec) positioned onto CA1 apical dendrites was flanked by two stimulating electrodes, S1 and S2, placed in the stratum radiatum (sr) layer to stimulate two independent Schaffer collateral (sc) synaptic inputs of the same neuronal population. **b** A weak tetanization (WTET, single train of 21 pulses at 100 Hz) applied to S2 (blue circle) induced a significant potentiation that gradually decayed to baseline level (*n* = 7 slices from 5 animals). The control input S1 (red circles) remained stable throughout the recording period. Strong-before-weak STC paradigm where a strong tetanization (STET, 3 trains of 100 pulses at 100 Hz, inter-trains interval of 10 min) was applied to S1 (red circles) followed by a WTET applied to S2 (blue circles) at various intervals, (**c**) 0.5 h (*n* = 8 slices from 5 animals), (**d**) 3 h (*n* = 7 slices from 6 animals), (**e**) 6 h (*n* = 7 slices from 7 animals), (**f**) 9 h (*n* = 7 slices from 5 animals). The LTPs induced in S1 and S2 maintained at potentiated level throughout the recording period in all cases. Analogue traces show typical fEPSP of S1 (red) and S2 (blue) at 15 min of baseline (dotted line), 10 min after LTP induction (dashed line), and at the end of the recording (solid line). Scale bar for all traces: 3 mV/5 ms. Three red arrows represent STET, whereas single blue arrow represents WTET. All data are represented as mean ± SEM.

Two-pathway experiments were performed in all experiments (Fig. 1a). Monopolar lacquer-coated stainless-steel electrodes (5 MΩ, AM Systems) were used in all cases. After the preincubation period, two stimulating electrodes, S1 and S2, were positioned at the stratum radiatum layer of the CA1 region to evoke field excitatory postsynaptic potential (fEPSP) at Schaffer collateral/commissural fibres–CA1 synapses. To obtain the fEPSP signal, a recording electrode was placed at the CA1 apical dendritic layer. The signals were amplified by a differential amplifier (Model 1700, AM Systems), digitized with an analogue-to-digital converter (Power 1401-3A, Cambridge Electronic Design) and monitored online using the Intracell software (Institute for Neurobiology, Magdeburg).

After positioning the electrodes, an input-output curve (fEPSP slope value vs afferent stimulation intensity) was plotted for each synaptic input. The test stimulation intensity was set to elicit a fEPSP slope response that was 40% of the maximal response obtained from the input-output curve. The recording was done at a 5-minute cycle. The average fEPSP slope value from 4 biphasic constant-current pulses at 0.2 Hz was used for each time point. A stable baseline of at least 30 min was recorded before LTP induction to assure neuronal metabolic stability^29^. If the fEPSP slope drifted by more than 10% during this period, new test pulse intensities were chosen, and the experiment was restarted. If the drift persisted, the experiment was abandoned. Additionally, experiments in which the control pathway fEPSP drifted by more than 10% within any one hour of the experiment were excluded. To induce LTP, either a strong tetanisation (STET) or weak tetanisation (WTET) protocol was used. STET consists of 3 stimulus trains of 100 pulses at 100 Hz (inter-trains interval of 10 min), while WTET consists of 1 stimulus train of 21 pulses at 100 Hz. Short-term potentiation (STP) was induced using a weaker tetanus stimulation consisting of a single train of 14 pulses^30^ or 11 pulses^3^ at 100 Hz.

### Statistics and reproducibility

All electrophysiological data were represented as mean ± standard error of mean (SEM). Each experimental group was represented by data from at least seven hippocampal slices (*n* ≥ 7) derived from a minimum of three biological replicates. The data of each experimental group was collected randomly from electrophysiological setups. The mean slope function of fEPSP at each time point (millivolts per millisecond) was calculated and normalised to the baseline value (averaged over 30 min stable baseline recording) following the convention that all brain slices count equal regardless of the absolute maximal slope value. Parametric test was used here though non-parametric test also yielded the same conclusion. To assess whether a LTP was maintained as a late-LTP or decayed back to baseline as an early-LTP, two-tailed paired t-test was used to compare the normalised fEPSPs between post-LTP time point (the last time point) and baseline (at 15th min of baseline). All graph plotting and statistical analyses were performed using Prism software (GraphPad). *p* < 0.05 was considered statistically significant^5^.

### Reporting summary

Further information on research design is available in the Nature Portfolio Reporting Summary linked to this article.

## Results

We first studied STC using the strong-before-weak paradigm. A WTET protocol induced an early-LTP that eventually returned to the baseline level (Fig. 1b; fEPSP_240min_ = 105.2 ± 2.4%, *p* = .1687). Pairing the WTET at 30 minutes after a STET produced a typical STC, with both synaptic inputs showing late-LTP (Fig. 1c; fEPSP_WTET__,__240 min_ = 151.5 ± 19.9%, *p* = 0.0332; fEPSP_STET__,__240 min_ = 171.1 ± 20.6%, *p* = 0.0121). Interestingly, when we increased the interval to 3 h, the WTET-LTP continued to transform into late-LTP (Fig. 1d; fEPSP_WTET__,__360 min_ = 140.6 ± 10.3%, *p* = 0.0069; fEPSP_STET__,__360 min_ = 156.4 ± 4.9%, *p* < 0.0001). This data suggests that the available pool of de novo PRPs are still interacting with the tags set by WTET to sustain the LTP. Therefore, we further extended the interval between STET and WTET to verify the effective duration for the occurrence of STC. Surprisingly, we found that both LTPs were sustained at a potentiated level even with interval of 6 h (Fig. 1e; fEPSP_WTET__,__540 min_ = 149.6 ± 15.9%, *p* = 0.0181; fEPSP_STET__,__540 min_ = 163.4 ± 8.5%, *p* = 0.0003) and 9 h (Fig. 1f; fEPSP_WTET__,__720 min_ = 149.1 ± 16.9%, *p* = 0.0286; fEPSP_STET__,__720 min_ = 136.1 ± 13.5%, *p* = 0.0422).

**Fig. 2:**
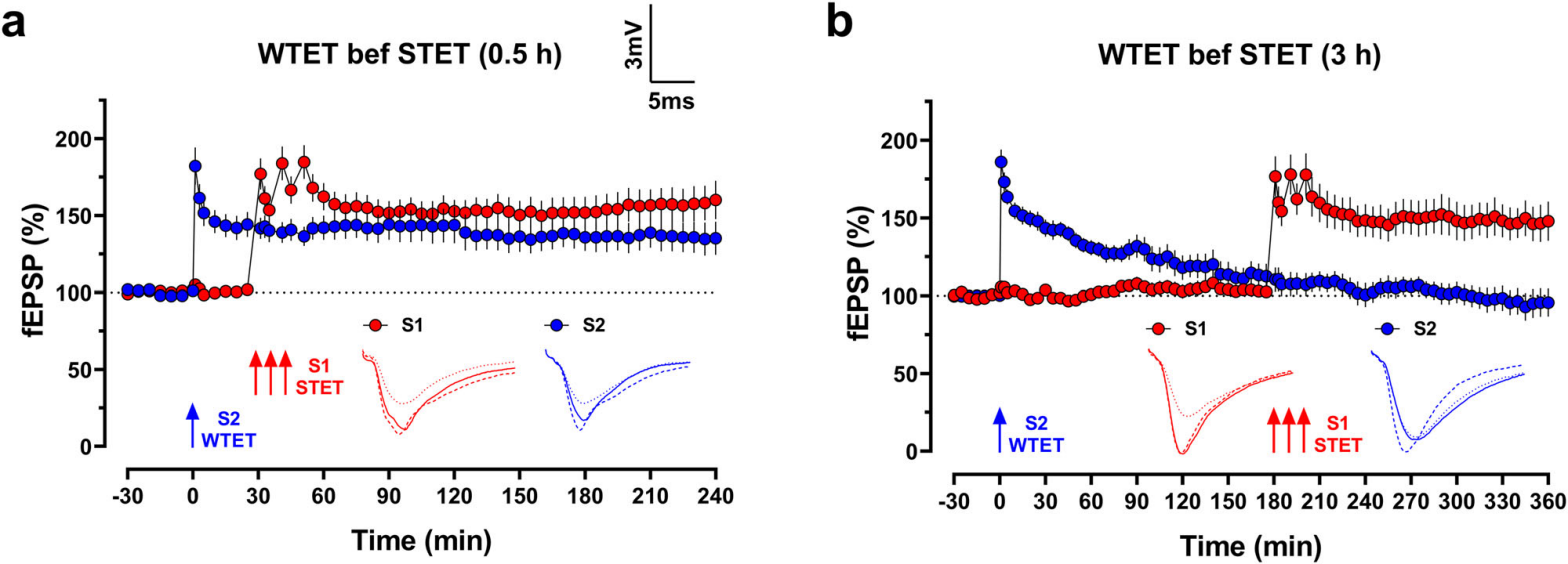
Extended interval in weak-before-strong synaptic tagging and capture paradigm fails to show associativity. **a** WTET applied to S2 (blue circles) followed by a STET applied to S1 (red circles) at a 0.5 h interval showed associativity where both LTPs sustained at potentiated level throughout the recording period (*n* = 7 slices from 5 animals). **b** When the interval between WTET and STET increased to 3 h, the WTET induced an early-form of LTP that gradually decayed to baseline level (*n* = 7 slices from 5 animals). Analogue traces show typical fEPSP of S1 (red) and S2 (blue) at 15 min of baseline (dotted line), 10 min after LTP induction (dashed line), and at the end of the recording (solid line). Scale bar for all traces: 3 mV/5 ms. Three red arrows represent STET, whereas a single blue arrow represents WTET. All data are represented as mean ± SEM.

Next, we employed the weak-before-strong paradigm to induce STC. Like the earlier results, a WTET induced on a synaptic input followed by a STET in another synaptic input at a 30-minute interval resulted in both the WTET- and STET-induced LTP maintained at potentiated level (Fig. 2a; fEPSP_WTET__,__240 min_ = 135.2 ± 10.8%, *p* = 0.0122; fEPSP_STET__,__240 min_ = 160.1 ± 12.4%, *p* = 0.0049). However, this associativity no longer existed when the STET was induced at 3 h after the WTET. Despite the STET-LTP remaining at a potentiated level throughout the recording, the WTET-LTP returned to baseline at the end of the recording (Fig. 2b; fEPSP_WTET__,__360 min_ = 95.6 ± 9.0%, *p* = 0.6420; fEPSP_STET__,__360 min_ = 148.0 ± 12.6%, *p* = 0.00061). Our results demonstrate that the effective tag setting duration to capture PRPs is less than 3 h, aligning with previous findings. However, the STC phenomenon could consistently occur if PRPs are readily available during the tag setting process, lasting at least 9 h in our ex vivo case.

**Fig. 3:**
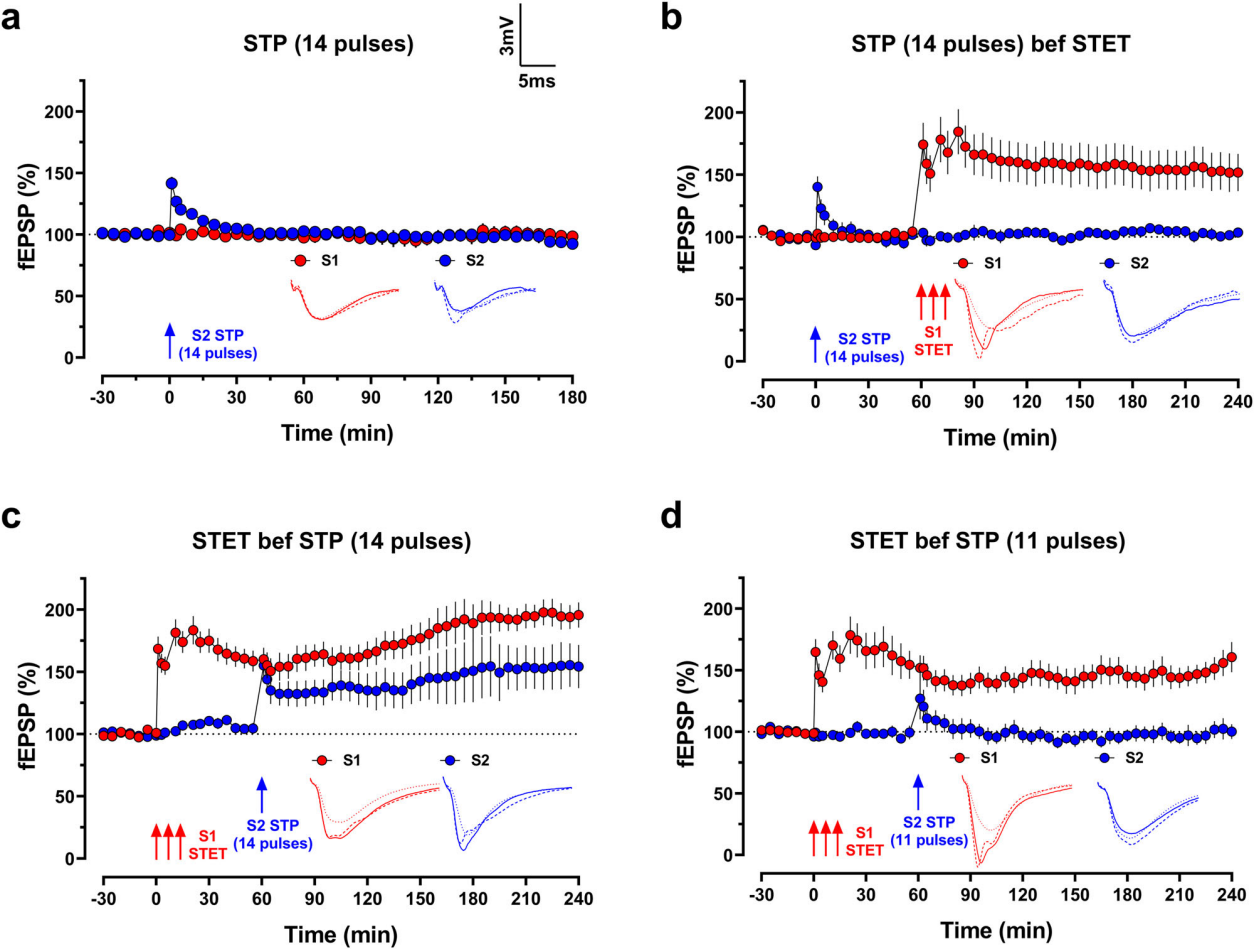
Strong tetanization lowers LTP induction threshold in neighbouring synapses. **a** A weak tetanus protocol (short-term potentiation (STP), 14 pulses at 100 Hz) applied to S2 (blue circles) induced a transient potentiation that returned to baseline level within 30 min (*n* = 10 slices from 5 animals). **b** STP (14 pulses) applied to S2 (blue circles) followed by STET applied to S1 (red circles) at a 1 h interval did not affect the STP-induced potentiation (*n* = 8 slices from 4 animals). **c** When STET was induced in S1 (red circles) followed by STP (14 pulses) in S2 (blue circles), the STP induced a long-lasting potentiation that sustained throughout the recording period (*n* = 7 slices from 3 animals). **d** Similar experiment as in **c**, except that the STP was replaced with an even weaker tetanus (11 pulses at 100 Hz). The STP-induced potentiation did not transform into a long-term potentiation in this case (*n* = 8 slices from 3 animals). Analogue traces show typical fEPSP of S1 (red) and S2 (blue) at 15 min of baseline (dotted line), 10 min after LTP induction (dashed line), and at the end of the recording (solid line). Scale bar for all traces: 3 mV/5 ms. Three red arrows represent STET, whereas single blue arrow represents STP (11 or 14 pulses). All data are represented as mean ± SEM.

We then hypothesised that the existing PRPs induced by STET could exert a metaplastic effect on the unstimulated synapses within the same neuronal population, thus lowering the threshold to induce LTP. Although Frey and Morris^3^ demonstrated that a single very weak tetanus (11 pulses at 100 Hz) following a strong tetanus in another input did not lead to STC, we predicted that this tetanus might be too weak to reach the lowered LTP threshold. Therefore, we used a separate weak tetanus protocol (STP, 14 pulses at 100 Hz) to induce a transient potentiation that returned to baseline within 30 min (Fig. 3a; fEPSP_180min_ = 92.5 ± 4.5%, *p* = 0.1152). Consistent with earlier studies^3,30^, STP (14 pulses) followed by STET at another synaptic input did not transform the transient potentiation into late-LTP (Fig. 3b; fEPSP_STP__,__240 min_ = 103.5 ± 3.7%, *p* = 0.3161; fEPSP_STET__,__240 min_ = 151.8 ± 14.9%, *p* = 0.0142). Intriguingly, when we reversed the order to STET preceding STP (14 pulses), the transient potentiation was now transformed into late-LTP (Fig. 3c; fEPSP_STP__,__240 min_ = 154.2 ± 17.3%, *p* = .0201; fEPSP_STET__,__240 min_ = 195.7 ± 10.2%, *p* < 0.0001). We also repeated Frey and Morris^3^ experiment and indeed confirmed that a weaker STP protocol with 11 pulses at 100 Hz did not result in STC (Fig. 3d; fEPSP_STP__,__240 min_ = 100.1 ± 6.0%, *p* = 0.8278; fEPSP_STET__,__240 min_ = 160.6 ± 12.2%, *p* = 0.0010), implying that the stimulation is too weak to reach the LTP threshold. These results suggest that the STP-inducing synaptic input has undergone metaplastic changes due to activity activated by STET in another synaptic input. Consequently, this enables a weaker stimulation to produce a late-LTP.

## Discussion

Previous studies have shown that “functional tags” last approximately 90 to 120 minutes to capture PRPs^3,6,31^. But what about the time window for the reverse scenario? How long can PRPs remain available for capture by later-set tags? This study explored the time course for effective associativity between different synaptic populations using ex vivo extracellular field electrophysiological recording techniques. The significant discovery is that tags set after an extended period (9 hours in our setting), following a strong tetanus in another synaptic input, still exhibit associativity. In addition to demonstrating the sharing of PRPs over an extended interval, we also identified a heterosynaptic metaplastic effect, in which a strong tetanus reduced the LTP threshold in neighbouring synaptic populations.

The extended duration of associativity observed in our strong-before-weak STC paradigm is notable, as homoeostatic mechanisms would be expected to counteract the PRP-sharing process over time. Frey and Morris^6^ proposed this possibility, though the precise time course has not been determined. At the behavioural level, this concept is supported by behavioural tagging models^32^. In these models, if the associative event (leading to long-term memory) precedes a weak learning task by 2–3 hours, the event loses its enhancing effect on the learning task^20,33–39^. The discrepancy between our findings and data from behavioural tagging models may stem from differences in the complexity of neuromodulatory systems between ex vivo and in vivo conditions. Neuromodulators such as acetylcholine are thought to play a role in homoeostatic plasticity, helping to prevent interference between previously stored memories and newly acquired information^40,41^. In ex vivo hippocampal slices, neuromodulation from extrahippocampal regions is absent, which may disrupt homoeostatic efficiency and contribute to the extended associativity duration observed in our study. However, the homoeostatic mechanisms underlying LTP maintenance and memory consolidation, as well as the role of neuromodulation in these processes, remain largely unexplored. Thus, while ex vivo electrophysiological recording is a valuable technique for identifying specific homoeostatic modulation pathways, in vivo studies are ultimately necessary to understand the coordinated, system-wide modulation. It is important to exercise considerable caution when translating cellular synaptic tagging and capture (STC) findings to behavioural tagging paradigms, as the interactions among neighbouring synapses and neurons at the cellular level may differ markedly from the interregional dynamics underlying behaviour.

Another mechanism that may facilitate the prolonged associativity is through heterosynaptic metaplasticity. Metaplasticity is the “plasticity of synaptic plasticity”, meaning that, synapses ability to undergo plastic changes can be influenced by its own prior events or even the activity of neighbouring synapses^42,43^. Our results indicate that a short-term potentiation (STP) protocol (14 pulses at 100 Hz), which typically induces transient potentiation, can lead to late-LTP when delivered after, but not before, a strong tetanus (STET) in close spatial and temporal proximity. This outcome could be explained in two ways. One possibility is that the STP protocol induces a brief tagging state (due to the weaker stimulation) that allows the transient potentiation to convert to late-LTP if PRPs are readily available during the tag-setting process. Alternatively, the STP protocol might enhance tag setting when applied after a STET, compared to its application prior to a STET, where fewer or no tags are established. This enhanced tag setting post-STET could facilitate the capture of PRPs, resulting in late-LTP. Indeed, previous studies have shown that LTP induction in one pathway can facilitate long-term depression in another pathway^44^. In another study, late-LTP induced in one synaptic input was shown to protect another synaptic input from depotentiation-induced LTP resetting^45^. Together, these findings support the latter explanation, suggesting that an STET metaplastically primes neighbouring synapses, facilitating future potentiation events. This may also contribute to the extended duration of associativity by priming the WTET-synapses to produce PRPs, however, this requires further validation. Nonetheless, regardless of the explanation, the weaker STP protocol (11 pulses at 100 Hz) used by Frey and Morris^3^, as well as in our experiments, did not result in late-LTP even when followed by an STET in a separate synaptic input. This suggests that the threshold for inducing tag setting, at least in our case, must exceed 11 pulses at 100 Hz. Interestingly, we previously reported that the threshold for setting a synaptic tag can be lowered by processes such as metaplasticity—specifically, ryanodine receptor activation before STP induction^30^. This enables tagging interactions in a weak-before-strong paradigm at 30 and 60 minutes but not at 90 minutes. In the current experiments, we used a 60-minute time window for STET-STP interactions and did not observe the expression of STC. However, it remains to be tested whether reducing the interval might promote the expression of STC. Additionally, the molecular mechanisms governing tag status and the time course of these metaplastic effects remain to be elucidated.

Studies on STC have proposed several molecules as potential tags, including Ca^2+^/calmodulin-dependent protein kinase II (CaMKII)^21^, protein kinase A^36,45^, and tropomyosin receptor kinase B (TrkB); however, the specific mechanisms of tag-PRP interaction remain largely unexplored. Notably, Sacktor^46^ proposed a model of synaptic autotagging by protein kinase M zeta (PKMζ) to explain the compartmentalization of activated PKMζ at specific synapses during the maintenance phase of LTP^46^. PKMζ is recognized as a key molecule for LTP maintenance and has been shown to persist at elevated levels in vivo for months, positioning it as a strong candidate for memory maintenance^47–49^. In the autotagging model, LTP induction activates PKMζ, increasing AMPAR levels at potentiated synapses. This elevation in GluR2 (an AMPAR subunit) serves as a tag to capture PKMζ during maintenance, establishing a cycle that sustains PKMζ activity at these synapses. Exploring the interaction between PKMζ and its substrates may enhance our understanding of tag-PRP interactions and provide insights into the mechanisms underlying synaptic associativity.

**Fig. 4:**
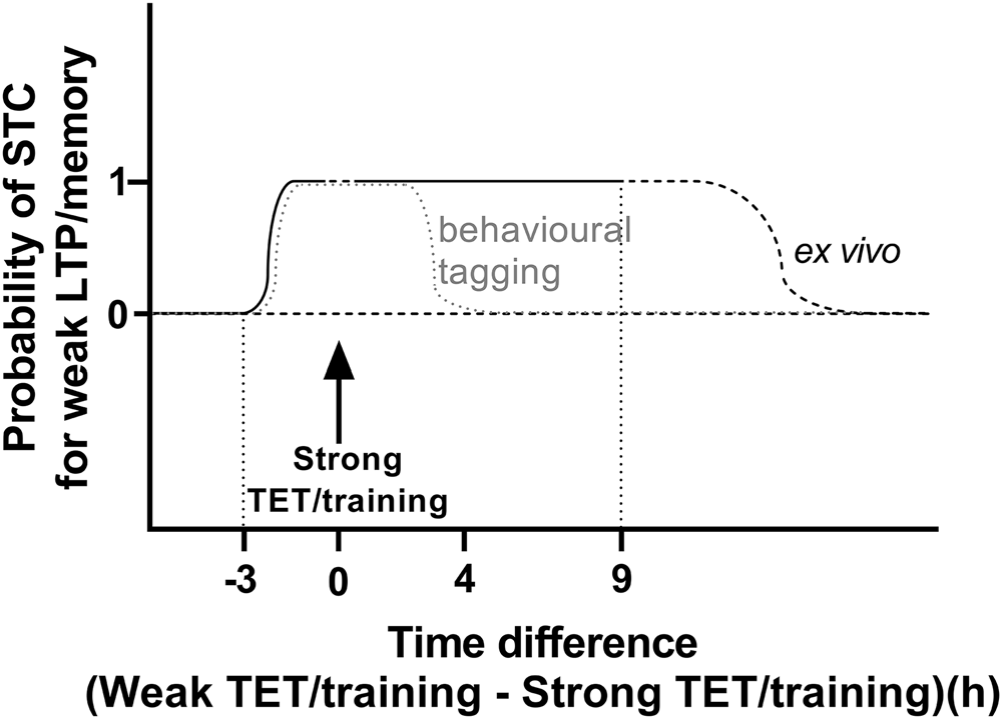
An illustration of the effective time window for synaptic/behavioural tagging and capture under behavioural tagging and ex vivo conditions. The arrow at 0 h time point indicates the timing where the strong TET/training is induced. Under ex vivo condition, no associativity is observed when weak TET precedes strong TET by 3 h. Associativity is observed for up to at least 9 h following strong TET induction. The effective associativity window is shorter under behavioural tagging conditions (dotted grey line)^32^. TET: tetanization.

How neurons regulate the time window of STC remains an open question. One hypothesis is that specific post-translational modifications of PRPs may limit further associativity beyond a certain period. This study suggests that investigating modifications of PRPs, such as PKMζ, in ex vivo versus in vivo conditions—particularly 4 hours after LTP induction (derived from behavioural tagging studies)—could shed light on these mechanisms (as illustrated in Fig. 4). The ex vivo hippocampal slices, which lack extrahippocampal homoeostatic modulation, may prevent PRP modifications that would otherwise restrict associativity. Targeting the mechanisms that delay the onset of synaptic homoeostasis—and thereby prolong the availability of plasticity-related proteins (PRPs) in behavioural paradigms that replicate the extended STC conditions—may represent a promising strategy to enhance associative learning. Elucidating the neuromodulatory processes and synaptic associativity underlying this phenomenon could provide critical insights into the molecular mechanisms of associative memory formation.

## Supplementary information

Reporting summary

## Data Availability

All data are available on the Open Science Framework (OSF) (https://osf.io/wqrk4/?view_only=750f66389bc2447686769ad9b9144fd8) and can be obtained from the corresponding author upon reasonable request.
